# Risk of Meningioma in European Patients Treated With Growth Hormone in Childhood: Results From the SAGhE Cohort

**DOI:** 10.1210/jc.2018-01133

**Published:** 2018-08-17

**Authors:** Anthony J Swerdlow, Rosie Cooke, Dominique Beckers, Gary Butler, Jean-Claude Carel, Stefano Cianfarani, Peter Clayton, Joël Coste, Annalisa Deodati, Emmanuel Ecosse, Anita C S Hokken-Koelega, Aysha J Khan, Wieland Kiess, Claudia E Kuehni, Christa E Flück, Roland Pfaffle, Lars Sävendahl, Grit Sommer, Muriel Thomas, Anders Tidblad, Sally Tollerfield, Gladys R J Zandwijken

**Affiliations:** 1 Institute of Cancer Research, Division of Genetics and Epidemiology, London, United Kingdom; 2 Institute of Cancer Research, Division of Breast Cancer Research, London, United Kingdom; 3 Unite d’Endocrinologie Pédiatrique, Centre Hospitalier Universitaire Université Catholique de Louvain Namur, Yvoir, Belgium; 4 Belgian Society for Pediatric Endocrinology and Diabetology, Brussels, Belgium; 5 University College London Institute of Child Health, London, United Kingdom; 6 University College London Hospitals National Health Service Foundation Trust, London, United Kingdom; 7 Assistance Publique-Hôpitaux de Paris, Hôpital Universitaire Robert-Debré, Department of Pediatric Endocrinology and Diabetology, Centre de Référence des Maladies Endocriniennes Rares de la Croissance, Paris, France; 8 Promoting Research Oriented Towards Early CNS Therapies, INSERM, Université Paris Diderot, Sorbonne Paris Cité, Paris, France; 9 Dipartimento Pediatrico Universitario Ospedaliero “Bambino Gesù” Children’s Hospital-Tor Vergata University, Rome, Italy; 10 Department of Women’s and Children’s Health, Karolinska Institutet, Stockholm, Sweden; 11 Royal Manchester Children’s Hospital, Central Manchester University Hospitals NHS Foundation Trust, Manchester Academic Health Science Centre, Manchester, United Kingdom; 12 Developmental Biology and Medicine, Faculty of Biology, Medicine, and Health, University of Manchester, Manchester, United Kingdom; 13 Biostatistics and Epidemiology Unit and Approches Psychologiques et Epidémiologiques des Maladies Chroniques Equipe d'Accueil, Paris, France; 14 Groupe Hospitalier Cochin-Saint Vincent de Paul and University Paris Descartes, Paris, France; 15 Dutch Growth Research Foundation, Rotterdam, Netherlands; 16 Erasmus Medical Center/Sophia Children’s Hospital, Rotterdam, Netherlands; 17 Hospital for Children and Adolescents and Centre of Pediatric Research, University of Leipzig, Leipzig, Germany; 18 Institute of Social and Preventive Medicine, University of Bern, Bern, Switzerland; 19 Division of Paediatric Endocrinology, Diabetology, and Metabolism, University Children’s Hospital Bern, Inselspital, Bern, Switzerland

## Abstract

**Context:**

There has been concern that GH treatment of children might increase meningioma risk. Results of published studies have been inconsistent and limited.

**Objective:**

To examine meningioma risks in relation to GH treatment.

**Design:**

Cohort study with follow-up via cancer registries and other registers.

**Setting:**

Population-based.

**Patients:**

A cohort of 10,403 patients treated in childhood with recombinant GH in five European countries since this treatment was first used in 1984. Expected rates from national cancer registration statistics.

**Main Outcome Measures:**

Risk of meningioma incidence.

**Results:**

During follow-up, 38 meningiomas occurred. Meningioma risk was greatly raised in the cohort overall [standardized incidence ratio (SIR) = 75.4; 95% CI: 54.9 to 103.6], as a consequence of high risk in subjects who had received radiotherapy for underlying malignancy (SIR = 658.4; 95% CI: 460.4 to 941.7). Risk was not significantly raised in patients who did not receive radiotherapy. Risk in radiotherapy-treated patients was not significantly related to mean daily dose of GH, duration of GH treatment, or cumulative dose of GH.

**Conclusions:**

Our data add to evidence of very high risk of meningioma in patients treated in childhood with GH after cranial radiotherapy, but suggest that GH may not affect radiotherapy-related risk, and that there is no material raised risk of meningioma in GH-treated patients who did not receive radiotherapy.

## Teaser

In a five-country cohort of 10,403 patients treated with recombinant growth hormone, meningioma risk was greatly raised in relation to radiotherapy, but not apparently related to growth hormone.

Since 1957 GH has been used to treat GH deficiency and short stature, initially using a human pituitary extract but since 1985 using solely recombinant GH (r-hGH).

GH causes increased serum concentrations of IGF-1. IGF-1 is antiapoptotic and mitogenic *in vitro*, and levels in adults have been associated in several studies with risks of subsequent malignancies (1). As a consequence, and because of early case reports and some findings in humans, there has been concern as to whether GH therapy might increase cancer risks (1, 2).

Meningiomas express GH receptors, and *in vitro* activation of the GH/IGF-1 axis increases the growth rate of meningiomas (3). In an *in vivo* model, downregulation of the GH/IGF-1 axis reduced meningioma growth (4). In the US Childhood Cancer Survivors Study cohort, second malignancy was significantly more common among GH-treated than non–GH-treated patients, and meningioma was much the most common second malignancy in the GH-treated group, accounting for 40% of all second neoplasms (5). A UK study (6) found meningiomas more common in GH-treated, brain-irradiated cancer patients than in matched, brain-irradiated cancer controls, but based on small numbers, and a later analysis from the US cohort did not find raised meningioma risk (7). The published results, however, have been based on relatively small numbers: 338 GH-treated patients in the US study (7) and 110 in the only other analysis, in the UK (6). To analyze the risk with much greater power, we therefore analyzed meningioma risks in the Safety and Appropriateness of Growth Hormone Treatments in Europe (SAGhE) study, a large cross-European cohort study of patients treated with r-hGH since 1984.

## Materials and Methods

The SAGhE study is a coordinated cohort study in eight European countries of patients treated with r-hGH at pediatric ages since such treatment was first used (1984 to 1986, depending on the country), and never treated with human pituitary GH. Details of the assembly of the cohort and methods of data collection have been described previously (8). Ethics committee agreement was obtained in every country, and for each patient either written informed consent was obtained, or the ethics committee stated that consent was not required. Only three patients in the cohort died of meningioma during follow-up, so we have only undertaken incidence analyses, not mortality analyses, for meningioma in this paper. Cancer incidence follow-up was via cancer registration and highly complete in Belgium, the Netherlands, Sweden, Switzerland, and the United Kingdom, and therefore analyses of incidence are restricted to these countries. The cohorts were national and population based, or virtually so, in Belgium, the Netherlands, Sweden, and the United Kingdom and clinic based and subnational in Switzerland. We obtained data on demographic and GH-related variables from existing databases and from case notes. Subjects were followed for mortality via national population-based registries in Belgium, the Netherlands, Sweden, and the United Kingdom, and by municipal registers and other means in Switzerland. In all countries, follow-up was independent of pharmaceutical companies. Vital status follow-up was highly complete. We excluded from analysis, individuals with certain conditions that both lead to GH therapy and are themselves very strong predisposing factors for malignancy [*e.g.*, type 1 neurofibromatosis, Fanconi syndrome (9)]. In addition, we also excluded from the cohort, subjects (n = 1) whose original diagnosis leading to GH treatment was meningioma.

We calculated person-years at risk for meningioma in the cohort by sex, 5-year age group, single calendar year, and country, commencing on the date of first treatment with GH and ending at whichever occurred earliest of: diagnosis of meningioma, death, loss to follow-up, or a fixed end date for each country (the date to which follow-up in that country was considered complete at the time the follow-up data were obtained). In Switzerland, cancer incidence follow-up was censored at age 16 or 21, depending on the canton, because cancer incidence data were from the Swiss Childhood Cancer Registry, which only covered these ages.

Meningiomas were taken as tumors coded to International Classification of Diseases 10 codes C70 (malignant), D32 (benign), and D42 (uncertain and unknown behavior) (10) and equivalents in International Classification of Diseases 9. Observed numbers of cancers and deaths in the cohort were compared with expectations derived from application of sex, age, country, and year-specific rates in the general population of each country to the person-years at risk in these categories in the cohort, to provide standardized incidence ratios (SIRs). Absolute excess rates were calculated by subtracting expected from observed numbers of cases, dividing by person-years at risk and multiplying by 10,000. Trends in risk with variables such as duration of GH treatment were tested as described by Breslow and Day (11); *P* values are all two sided.

As well as analyses of risks in the cohort overall, we also analyzed the data in subdivisions by initial diagnosis, whether radiotherapy was received, and cumulative dose, mean daily dose, and duration of GH treatment. To be able to explore potential surveillance bias in the diagnosis of meningiomas in the cohort, we endeavored to discover from clinical sources for each UK patient, the pathway that had led to diagnosis of the meningioma.

## Results

Of 10,786 patients recorded as treated with r-hGH in the five study countries, 257 had to be excluded from analysis because of lack of permission for cancer incidence follow-up or lack of data, and 126 because of an underlying diagnosis at high risk of cancer or an underlying diagnosis of meningioma as the reason for GH treatment. This left 10,403 who formed the study cohort. Just over one-half were male and four-fifths were aged 5 to 14 years at first treatment (Table 1). The most common underlying diagnoses were isolated growth failure (n = 3952), and malignancy (n = 1830).

**Table 1.** Descriptive Characteristics of Patients in the SAGhE Cohort Followed for Risk of Meningioma

Characteristic	No.	%
Sex		
Male	5530	53.2
Female	4873	46.8
Country		
Belgium	1325	12.7
Netherlands	1685	16.2
Sweden	2822	27.1
Switzerland	737	7.1
United Kingdom	3834	36.9
Age started GH treatment, y		
0–4	1130	10.9
5–9	3632	34.9
10–14	4834	46.5
15–19	807	7.8
Year started GH treatment		
<1990	2070	19.9
1990–1994	3976	38.2
1995–1999	2840	27.3
≥2000	1517	14.6
Diagnosis leading to GH treatment		
CNS tumor	1307	12.6
Non-CNS solid tumor	97	0.9
Hematological malignancy	426	4.1
Chronic renal failure and renal diseases	139	1.3
Turner syndrome	1721	16.5
Other syndromes and chronic diseases	1003	9.6
Multiple pituitary hormone deficiency organic	1343	12.9
Skeletal dysplasias	286	2.8
Isolated growth failure^*a*^	3952	38.0
Nonclassifiable	129	1.2
Total	10,403	100.0

^a^ Including isolated GH deficiency, idiopathic short stature, and small for gestational age.

During follow-up, 326 patients died, 175 were lost to follow-up, 38 were diagnosed with meningioma (30 benign, one malignant, and seven of uncertain behavior), and 9864 survived without meningioma to the end of the follow-up period. A total of 154,795 person-years at risk were accrued, an average of 14.9 years per patient. The SIR for meningioma in the cohort overall was 75.4 (95% CI: 54.9 to 103.6) (Table 2), and the absolute excess rate was 2.4 per 10,000 (not in Table). Relative risks were similar in males and females, and greatly raised in the Netherlands, Sweden, and the United Kingdom. There were no cases in Belgium and Switzerland but expected numbers were small (0.04 and 0.01, respectively) and 95% CIs included the all-country SIR. All but one of the meningiomas occurred in patients whose initial diagnosis was cancer [SIR = 466.3 (95% CI: 337.8 to 643.5)]; the risk was not significantly raised in patients whose initial diagnosis was not cancer [SIR = 2.4 (95% CI: 0.3 to 16.7)]. Risks were over 300-fold raised for patients whose initial diagnoses were central nervous system (CNS) tumor; hematological malignancy; or non-CNS solid tumor (Table 2).

**Table 2.** Risk of Meningioma in the Cohort in Relation to Sex, Country of Residence, and Initial Diagnosis Leading to GH Treatment

	All Initial Diagnoses		Initial Diagnosis Cancer		Initial Diagnosis Noncancer	
	n	SIR (95% CI)	n	SIR (95% CI)	n	SIR (95% CI)
Sex						
Male	18	83.7 (52.7, 132.8)^*a*^	18	464.9 (292.9, 737.8)^*a*^	0	0.0 (0.0, 20.5)
Female	20	69.2 (44.7, 107.3)^*a*^	19	467.6 (298.3, 733.1)^*a*^	1	4.0 (0.6, 28.6)
Country of residence						
Belgium	0	0.0 (0.0, 92.2)	0	0.0 (0.0, 368.9)	0	0.0 (0.0, 92.2)
Netherlands	9	84.4 (43.9, 162.2)^*a*^	9	503.4 (261.9, 967.5)^*a*^	0	0.0 (0.0, 41.0)
Sweden	7	40.5 (19.3, 85.0)^*a*^	7	385.6 (183.8, 808.8)^*a*^	0	0.0 (0.0, 24.6)
Switzerland	0	0.0 (0.0, 368.9)	0	0.0 (0.0, 6148.1)	0	0.0 (0.0, 368.9)
United Kingdom	22	126.8 (83.5, 192.6)^*a*^	21	593.5 (387.0, 910.3)^*a*^	1	7.2 (1.0, 51.4)
Diagnosis leading to GH treatment						
CNS tumor	29	533.7 (370.9, 768.0)^*a*^	29	533.7 (370.9, 768.0)^*a*^	—	—
Hematological malignancy	7	319.2 (152.2, 669.5)^*a*^	7	319.2 (152.2, 669.5)^*a*^	—	—
Non-CNS solid tumor	1	324.1 (45.6, 2300.6)^*b*^	1	324.1 (45.6, 2300.6)^*b*^	—	—
Turner syndrome	1	9.2 (1.3, 65.0)^*c*^	—	—	1	9.2 (1.3, 65.0)^*c*^
Isolated growth failure	0	0.0 (0.0, 19.4)	—	—	0	0.0 (0.0, 19.4)
Other noncancer	0	0.0 (0.0, 30.7)	—	—	0	0.0 (0.0, 30.7)
Total	38	75.4 (54.9, 103.6)^*a*^	37	466.3 (337.8, 643.5)^*a*^	1	2.4 (0.3, 16.7)

^a^ *P* < 0.001.

^b^ *P* < 0.01.

^c^ *P* < 0.05.

We had information that 1178 of the patients had received cranio(-spinal) radiotherapy (all but 13 for cancer) and 3055 had not received cranio(-spinal) radiotherapy, and for 6170, this was not known. Thirty of the 38 meningiomas occurred in the cancer patients known to have received cranio(-spinal) radiotherapy (Table 3). The relative risk of meningioma for cancer patients treated with radiotherapy was over 600 (Table 3). The SIR was not related to age at first GH treatment, time since starting treatment, or attained age. There were also no significant trends in risk with mean daily GH dose, duration of treatment, or cumulative dose of GH. Of the remaining meningioma cases, seven occurred in patients with unknown radiotherapy status [SIR = 277.5 (95% CI: 132.3 to 582.1)]; all were in Sweden, for which the databases used for this study did not include data on radiotherapy to allow them to be included in risk analyses, but on separate inquiry, four had received prior radiotherapy, and for three, no information on this was available. One meningioma occurred among patients without radiotherapy (a patient with Turner syndrome), for whom risk was not significantly raised.

**Table 3.** Risk of Meningioma in Patients Whose Initial Diagnosis Was Cancer and Were Treated by Radiotherapy, by Age and GH Treatment Variables

	n	SIR (95% CI)
Age started GH treatment, y		
0–4	1	1401.5 (197.4, 9949.0)^*a*^
5–9	9	782.4 (407.1, 1503.7)^*b*^
10–14	19	644.7 (411.2, 1010.7)^*b*^
15-19	1	258.1 (36.4, 1832.1)^*a*^
* P* trend		0.21
Time since started GH treatment, y		
0–4	2	338.0 (84.5, 1351.4)^*b*^
5–9	2	197.5 (49.4, 789.5)^*b*^
10–14	14	1130.7 (669.7, 1909.2)^*b*^
15–19	10	857.0 (461.1, 1592.8)^*b*^
≥20	2	365.8 (91.5, 1462.5)^*b*^
* P* trend		0.26
Attained age, y		
0–9	0	0.0 (0.0, 12,296.3)
10–19	6	487.2 (218.9, 1084.3)^*b*^
20–29	21	863.5 (563.0, 1324.4)^*b*^
≥30	3	346.7 (111.8, 1074.8)^*b*^
* P* trend		0.95
Duration of GH treatment, y		
<3	8	547.5 (273.8, 1094.7)^*b*^
3–5	11	587.3 (325.3, 1060.5)^*b*^
≥6	11	998.9 (553.2, 1803.8)^*b*^
* P* trend		0.19
Mean GH dose, µg/kg/d		
<20	7	635.1 (302.8, 1332.2)^*b*^
20–29	17	805.4 (500.7, 1295.6)^*b*^
30–39	3	425.1 (137.1, 1318.1)^*b*^
≥40	1	1297.5 (182.8, 9210.9)^*a*^
* P* trend		0.92
Cumulative GH dose, mg/kg		
<25	8	511.9 (256.0, 1023.7)^*b*^
25–49	10	601.3 (323.6, 1117.6)^*b*^
50–99	11	1286.0 (712.2, 2322.1)^*b*^
≥100	0	0.0 (0.0, 4098.8)
* P* trend		0.13
Total	30	658.4 (460.4, 941.7)^*b*^

^a^ *P* < 0.01.

^b^ *P* < 0.001.

^c^ *P* < 0.05.

Of the 22 meningiomas diagnosed in patients in the United Kingdom, we were able to obtain information on the events leading to diagnosis for 14; of these, nine were diagnosed after symptomatic presentations and five at routine follow-up.

## Discussion

Our analysis of over 10,000 patients treated with GH in childhood showed meningioma risk over 70-fold, highly significantly, raised in this cohort compared with general population expectations. This was a consequence of a risk six times greater than this in the subset of patients who had received GH after treatment of cancer, and within these, greater risk again in the patients who had received cranio(-spinal) radiotherapy. Although we do not have data on radiotherapy dose, incidence of GH deficiency after cranial radiotherapy is dose and time dependent (12–14), and most of the cancer patients had brain tumors, which are usually treated with 40 to 50 Gy (12), so we would expect that radiotherapy doses in the cohort will generally have been ≥40 Gy.

The relative risks in our cohort for meningioma are far larger than for any other tumor after GH treatment (9). Because ionizing radiation exposure is a well-established cause of meningioma (15, 16), including after radiation therapy of childhood cancers (17, 18), the extraordinarily large risk in our GH-treated cohort does not in itself incriminate GH. Comparisons of follow-up of GH-treated and untreated cancer patients in the United States and United Kingdom (5, 6) have given some evidence of raised risk of meningioma associated with GH, although a later analysis from the US cohort (7) did not find raised risk. Our study had the weakness that we were not able to compare risks in our GH cohort directly with untreated patients, because we did not have data on such patients. On the other hand, our study had the strength that we were able to, unlike previous studies, analyze risks in relation to dose and duration of GH treatment—critical variables in assessing whether there is an etiological relationship (19). These GH variables were not significantly related to meningioma risk and furthermore there was no significant raised risk of meningioma in the 8573 noncancer patients in our cohort who received GH therapy. Thus our data, based on different variables and a far larger cohort than previously, do not support the hypothesis that GH treatment influences meningioma risk. We were not able to collect IGF-1 data for the cohort, but future research would be improved by investigating, if practical, whether IGF-1 levels during GH treatment relate to subsequent meningioma risk. We were also not able to analyze meningioma risks in relation to extent of, or treatment of, other pituitary deficiencies, but these seem unlikely to explain the meningioma risk in these patients because the majority of cases did not have a record of other pituitary deficiencies and only 13 had a record of treatment of such deficiencies.

The main reason for the raised meningioma risk in the cohort is likely to be ionizing radiation exposure. Previous cohort studies of meningioma risk after radiation exposure have found excess relative risks per Gy ranging from 0.64 to 5.1, with a summary excess relative risk across studies of 1.81 (16). Our relative risks are of the same order as those for ≥40 Gy exposures to the meninges in a large UK childhood cancer cohort (17), but several times larger than those found in a similar US cohort (18).

Meningioma is a tumor for which there is known to be a high prevalence of subclinical disease: on brain MRI in the general population, 0.5% of individuals aged 45 to 59 (the youngest ages studied) had incidental findings of meningioma (20). There is therefore considerable scope for intensive medical contacts and cerebral imaging (especially MRI) consequent on underlying cerebral malignancies and GH treatment in our cohort to lead to diagnosis of asymptomatic meningiomas that would not otherwise have been detected, or at least not at that time. Such a “screening” effect, if there is one, might be expected to operate particularly around (or indeed before) the time of first treatment with GH, when prevalent asymptomatic meningiomas incident over many years previously might come to light, and to diminish subsequently, when only newly incident cases would be available for detection. Our data, however, did not show diminishing risks with longer time since first treatment. Furthermore, among the UK cases for whom we could identify the pathway to diagnosis, most of the tumors were investigated because of symptoms (although we cannot tell, of course, whether these symptoms would not have been presented, or not have been investigated further, if the patient had not had a previous cerebral tumor and GH treatment).

A subtler screening effect might have occurred if improvements in imaging technology over time had caused detection of some meningiomas in the cohort in recent years that were already present but undetected at the time of earlier, lower sensitivity imaging (6). This could have led to artifactual raised risks throughout follow-up; we do not have data to measure the extent, if any, of such an effect.

In conclusion, our data add to evidence of the very high relative risks of meningioma in patients treated in childhood with r-hGH after cranial radiotherapy for malignancy. Clinically it is important to be aware of this risk when following up such patients. Our data and the previous literature on radiation effects indicate that the raised risk is mainly due to radiotherapy, although it may also to some extent reflect detection of asymptomatic meningiomas as a consequence of intensive medical surveillance and cerebral imaging in these patients. Our data also suggest, however, that GH treatment has not augmented further the radiotherapy-related risk.

## Abbreviations:

CNS: central nervous system
r-hGH: recombinant GH
SAGhE: Safety and Appropriateness of Growth Hormone Treatments in Europe
SIR: standardized incidence ratio

## References

[1] Clayton PE, Banerjee I, Murray PG, Renehan AG Growth hormone, the insulin-like growth factor axis, insulin and cancer risk. Nat Rev Endocrinol. 2011;7(1):11–24. 20956999 10.1038/nrendo.2010.171

[2] Swerdlow AJ Does growth hormone therapy increase the risk of cancer? Nat Clin Pract Endocrinol Metab. 2006;2(10):530–531. 17024146 10.1038/ncpendmet0295

[3] Friend KE, Radinsky R, McCutcheon IE Growth hormone receptor expression and function in meningiomas: effect of a specific receptor antagonist. J Neurosurg. 1999;91(1):93–99. 10389886 10.3171/jns.1999.91.1.0093

[4] McCutcheon IE, Flyvbjerg A, Hill H, Li J, Bennett WF, Scarlett JA, Friend KE Antitumor activity of the growth hormone receptor antagonist pegvisomant against human meningiomas in nude mice. J Neurosurg. 2001;94(3):487–492. 11235955 10.3171/jns.2001.94.3.0487

[5] Ergun-Longmire B, Mertens AC, Mitby P, Qin J, Heller G, Shi W, Yasui Y, Robison LL, Sklar CA Growth hormone treatment and risk of second neoplasms in the childhood cancer survivor. J Clin Endocrinol Metab. 2006;91(9):3494–3498. 16822820 10.1210/jc.2006-0656

[6] Mackenzie S, Craven T, Gattamaneni HR, Swindell R, Shalet SM, Brabant G Long-term safety of growth hormone replacement after CNS irradiation. J Clin Endocrinol Metab. 2011;96(9):2756–2761. 21715535 10.1210/jc.2011-0112

[7] Patterson BC, Chen Y, Sklar CA, Neglia J, Yasui Y, Mertens A, Armstrong GT, Meadows A, Stovall M, Robison LL, Meacham LR Growth hormone exposure as a risk factor for the development of subsequent neoplasms of the central nervous system: a report from the childhood cancer survivor study. J Clin Endocrinol Metab. 2014;99(6):2030–2037. 24606096 10.1210/jc.2013-4159 PMC4037726

[8] Swerdlow AJ, Cooke R, Albertsson-Wikland K, Borgström B, Butler G, Cianfarani S, Clayton P, Coste J, Deodati A, Ecosse E, Gausche R, Giacomozzi C, Kiess W, Hokken-Koelega AC, Kuehni CE, Landier F, Maes M, Mullis PE, Pfaffle R, Sävendahl L, Sommer G, Thomas M, Tollerfield S, Zandwijken GR, Carel JC Description of the SAGhE cohort: A large European study of mortality and cancer incidence risks after childhood treatment with recombinant growth hormone. Horm Res Paediatr. 2015;84(3):172–183. 26227295 10.1159/000435856 PMC4611066

[9] Swerdlow AJ, Cooke R, Beckers D, Borgström B, Butler G, Carel JC, Cianfarani S, Clayton P, Coste J, Deodati A, Ecosse E, Gausche R, Giacomozzi C, Hokken-Koelega ACS, Khan AJ, Kiess W, Kuehni CE, Mullis PE, Pfaffle R, Sävendahl L, Sommer G, Thomas M, Tidblad A, Tollerfield S, Van Eycken L, Zandwijken GRJ Cancer risks in patients treated with growth hormone in childhood: the SAGhE European cohort study. J Clin Endocrinol Metab. 2017;102(5):1661–1672. 28187225 10.1210/jc.2016-2046 PMC6061931

[10] World Health Organization International Statistical Classification of Diseases and Related Health Problems. 10th Revision. Geneva, Switzerland: World Health Organization; 1992.

[11] Breslow NE, Day NE Statistical methods in cancer research. Volume I - The analysis of case-control studies. IARC Sci Publ. 1980; (32):5–338. 7216345

[12] Darzy KH Radiation-induced hypopituitarism after cancer therapy: who, how and when to test. Nat Clin Pract Endocrinol Metab. 2009;5(2):88–99. 19165221 10.1038/ncpendmet1051

[13] Clayton PE, Shalet SM Dose dependency of time of onset of radiation-induced growth hormone deficiency. J Pediatr. 1991;118(2):226–228. 1993949 10.1016/s0022-3476(05)80487-1

[14] Rappaport R, Brauner R Growth and endocrine disorders secondary to cranial irradiation. Pediatr Res. 1989;25(6):561–567. 2662128 10.1203/00006450-198906000-00001

[15] Wiemels J, Wrensch M, Claus EB Epidemiology and etiology of meningioma. J Neurooncol. 2010;99(3):307–314. 20821343 10.1007/s11060-010-0386-3 PMC2945461

[16] Braganza MZ, Kitahara CM, Berrington de González A, Inskip PD, Johnson KJ, Rajaraman P Ionizing radiation and the risk of brain and central nervous system tumors: a systematic review. Neuro-oncol. 2012;14(11):1316–1324. 22952197 10.1093/neuonc/nos208 PMC3480263

[17] Taylor AJ, Little MP, Winter DL, Sugden E, Ellison DW, Stiller CA, Stovall M, Frobisher C, Lancashire ER, Reulen RC, Hawkins MM Population-based risks of CNS tumors in survivors of childhood cancer: the British Childhood Cancer Survivor Study. J Clin Oncol. 2010;28(36):5287–5293. 21079138 10.1200/JCO.2009.27.0090 PMC4809645

[18] Neglia JP, Robison LL, Stovall M, Liu Y, Packer RJ, Hammond S, Yasui Y, Kasper CE, Mertens AC, Donaldson SS, Meadows AT, Inskip PD New primary neoplasms of the central nervous system in survivors of childhood cancer: a report from the Childhood Cancer Survivor Study. J Natl Cancer Inst. 2006;98(21):1528–1537. 17077355 10.1093/jnci/djj411

[19] Hill AB The environment and disease: association or causation? Proc R Soc Med. 1965;58:295–300. 14283879 10.1177/003591576505800503 PMC1898525

[20] Vernooij MW, Ikram MA, Tanghe HL, Vincent AJ, Hofman A, Krestin GP, Niessen WJ, Breteler MM, van der Lugt A Incidental findings on brain MRI in the general population. N Engl J Med. 2007;357(18):1821–1828. 17978290 10.1056/NEJMoa070972

